# Human Impacts on the Vegetation of the Juan Fernández (Robinson Crusoe) Archipelago

**DOI:** 10.3390/plants12234038

**Published:** 2023-11-30

**Authors:** Tod F. Stuessy, Daniel J. Crawford, Josef Greimler

**Affiliations:** 1 Herbarium and Department of Evolution, Ecology, and Organismal Biology, The Ohio State University, 1315 Kinnear Road, Columbus, OH 43212, USA; 2 Department of Botany and Biodiversity Research, University of Vienna, Rennweg 14, A-1030 Vienna, Austria; josef.greimler@univie.ac.at; 3 Department of Ecology and Evolutionary Biology and the Biodiversity Institute, University of Kansas, 1200 Sunnyside Avenue, Lawrence, KS 66045, USA; dcrawfor@ku.edu

**Keywords:** conservation, evolution, genetic variation, historical ecology, islands

## Abstract

The human footprint on marine and terrestrial ecosystems of the planet has been substantial, largely due to the increase in the human population with associated activities and resource utilization. Oceanic islands have been particularly susceptible to such pressures, resulting in high levels of loss of biodiversity and reductions in the numbers and sizes of wild populations. One archipelago that has suffered from human impact has been the Juan Fernández (Robinson Crusoe) Archipelago, a Chilean national park located 667 km west of Valparaíso at 33° S. latitude. The park consists of three principal islands: Robinson Crusoe Island (48 km^2^); Santa Clara Island (2.2 km^2^); and Alejandro Selkirk Island (50 km^2^). The latter island lies 181 kms further west into the Pacific Ocean. No indigenous peoples ever visited or lived on any of these islands; they were first discovered by the Spanish navigator, Juan Fernández, in 1574. From that point onward, a series of European visitors arrived, especially to Robinson Crusoe Island. They began to cut the forests, and such activity increased with the establishment of a permanent colony in 1750 that has persisted to the present day. Pressures on the native and endemic flora increased due to the introduction of animals, such as goats, rats, dogs, cats, pigs, and rabbits. Numerous invasive plants also arrived, some deliberately introduced and others arriving inadvertently. At present, more than three-quarters of the endemic and native vascular species of the flora are either threatened or endangered. The loss of vegetation has also resulted in a loss of genetic variability in some species as populations are reduced in size or go extinct. It is critical that the remaining genetic diversity be conserved, and genomic markers would provide guidelines for the conservation of the diversity of the endemic flora. To preserve the unique flora of these islands, further conservation measures are needed, especially in education and phytosanitary monitoring.

## 1. Introduction

In the Anthropocene, it is difficult to find an area of the planet that has not been disturbed by humans. Our species, largely due to population increases, geographical expansion, and resource utilization, has impacted all known ecological zones [1,2,3,4] on all continents, including Antarctica [5]. Impacts have also been felt in the marine environment [6,7], especially through overfishing and contamination from plastics and toxic waste [8,9], plus recent global warming [10].

Of all the areas on Earth, oceanic islands have been particularly vulnerable to human disturbance. These small land masses, surrounded by water, have been visited and colonized by people for millennia from two fundamental levels: (1) peoples expanding and colonizing new islands, such as the waves of Polynesians throughout the Pacific [11]; and (2) the arrival of European explorers from England, France, Italy, the Netherlands, Portugal, and Spain. Many oceanic islands lie in tropical or subtropical zones, where attractive beaches have drawn tourists. To attend to these visitors, airports, hotels, roads, houses, hospitals, plantations, and animal ranches have been developed. The island of Oahu in the Hawaiian Archipelago, with the bustling city of Honolulu and its massive tourist industry, serves as one example.

The influx of humans and their activities on oceanic islands has resulted in conflict with indigenous wildlife that has evolved there over millions of years. Isolated islands, often far remote from immigrant source areas, have evolved high levels of endemic biotas of high value for understanding biogeographic origins and patterns and processes of evolution, hence serving as natural laboratories for studying organic evolution [12]. Levels of endemism in some animal groups can be extremely high, such as in *Drosophila* in the Hawaiian Islands with more than 600 endemic species [13]. Specific endemism among plant groups can also be high, approaching 90% of the vascular flora of the Hawaiian Archipelago [12]. Divergence in oceanic islands has ensued in some cases to the extent of the taxonomic recognition of distinct island genera or even families.

Ongoing global warming will affect the adaptation potential of oceanic islands depending on their size and physical features [10], especially impacting floras of small islands due to their often small populations of endemics [14]. For the Canary Islands, Hanz et al. [15] (p. 1167) conclude “…that a large proportion of [their] flora could be able to cope with the predicted climatic changes. Nonetheless, with ongoing climate change, a net loss of species with unique functions seems inevitable, leading to functional homogenization, and impoverishment, and a possible deterioration of ecosystem stability.”

**Figure 1:**
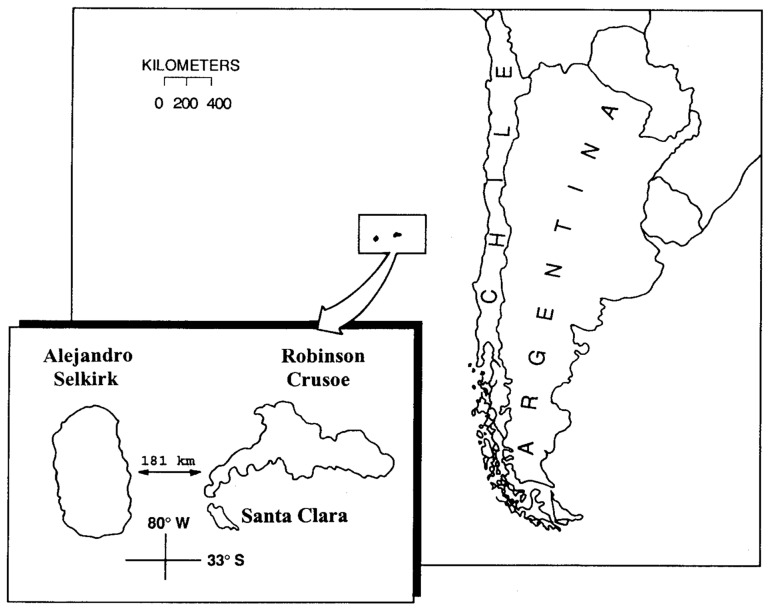
Map of the Juan Fernández (Robinson Crusoe) Archipelago in the eastern Pacific.

An oceanic archipelago of particular interest for understanding the effects of human interventions is the Juan Fernández (Robinson Crusoe) Archipelago. This island group consists of three major islands (Figure 1): (1) Robinson Crusoe Island (48 km^2^); (2) Santa Clara Island (2.2 km^2^), off the southwestern coast of Robinson Crusoe Island; and (3) Alejandro Selkirk Island (50 km^2^). The islands are known to be different radiometric ages with Robinson Crusoe and Santa Clara about 4 myr and Alejandro Selkirk c. 1 myr [16,17]. The number of endemic vascular species is 129, with 104 angiosperms and 25 ferns, and with a level of specific endemism of 64% [18]. Eleven endemic genera also occur, including the endemic monospecific families Thyrsopteridaceae (fern) and Lactoridaceae (flowering plant).

No evidence exists of human groups ever having arrived at the Juan Fernández Islands [19]. The archipelago was free of human influence prior to 1574 when Robinson Crusoe Island (Más a Tierra) was discovered by the Spanish navigator Juan Fernández. From this point forward, humans visited the island, and their activities began to impact the vegetation. Accompanying these visits have been extensive documentations of what was conducted, when, and where. Captains were required to keep logs of their activities, in considerable detail, and these serve as guides to the human impacts on vegetation. Many other visitors also came to the islands, especially to Robinson Crusoe Island with its natural harbor for safe anchorage, and they published accounts of their observations of the landscape. Much of this historical documentation has recently been synthesized and evaluated [17].

As humans altered the island forests on Robinson Crusoe Island, they also impacted the number and size of populations of the endemic plant species. As populations decreased in number, this led to a reduction in the genetic diversity in many species. Although some endemics on Robinson Crusoe Island still maintain sizeable populations and high levels of genetic diversity (e.g., *Nothomyrcia fernandeziana*) [20], other species have been greatly reduced, resulting in a negative conservation status as threatened, endangered, or critically endangered [21,22]. Species containing low levels of genetic variation are much more susceptible to environmental perturbations because they cannot adapt to changes in the habitat and hence may deserve higher conservation priority.

The purposes of this paper are to (1) review the present state of vegetation of the islands of the Juan Fernández Archipelago; (2) summarize the historical record of human contacts with the islands and their impacts on the vegetation; (3) demonstrate patterns of genetic variation among populations of endemic species that have resulted from combined natural and human disturbances; and (4) offer recommendations for further conservation of the flora and vegetation.

## 2. Present State of Vegetation

A number of previous descriptive investigations on the vegetation of the Juan Fernández Archipelago have been published over the past two centuries, beginning with Gay in 1832 [23], Hemsley in 1884 [24], followed by Johow in 1896 [25], and then Skottsberg in the first decades of the 20th century [26]. Kunkel in 1957 [27] provided an assessment of the vegetation on the top of El Yunque, the tallest peak on Robinson Crusoe Island, Schwaar in 1979 [28] produced a transect on Robinson Crusoe Island, and Nishida and Nishida in 1981 [29] added additional observations. A very comprehensive effort was completed by Ortíz-Riveros and co-workers in 1982 [30], but this resulted in a very complex system with 120 different vegetation categories, too dissected for seeking correlations with biogeographic and evolutionary concepts within and between islands. Danton more recently [31] added additional commentary about the native “myrtisylve” forests in the archipelago.

**Figure 2:**
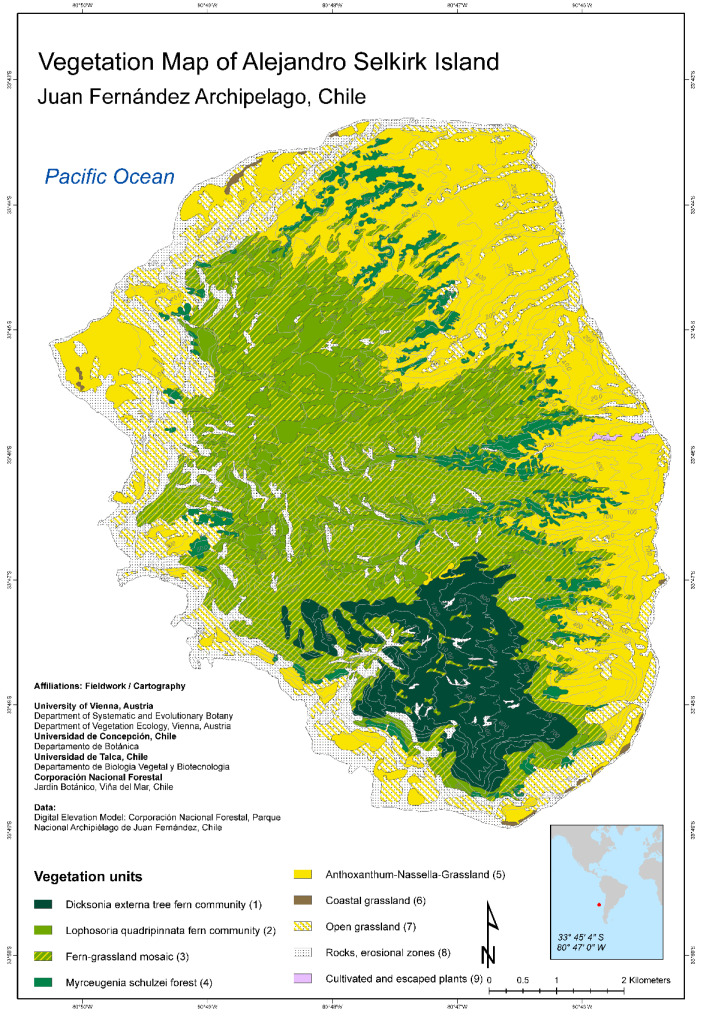
Vegetation map of Alejandro Selkirk Island [33].

**Figure 3:**
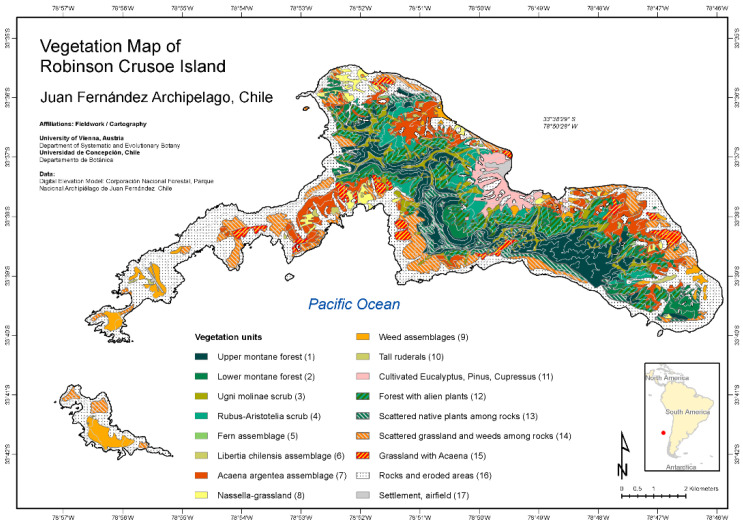
Vegetation map of Robinson Crusoe Island [32] (modified).

New vegetation maps for all three islands of the Juan Fernández Archipelago have been published recently by Josef Greimler and colleagues ([32,33] Figure 2 and Figure 3). These maps were based on available topographic maps for the islands, aerial photographs taken by the Chilean Airforce in 1980 for Robinson Crusoe Island, Bing images taken in 2001 and 2004 provided by ESRI-basemaps for Alejandro Selkirk Island, and ground observations and accompanying photographs from expeditions in 1999, 2000, and 2011. The principal method of documentation of species and their dominance and abundance was with relevés, a technique pioneered by Braun-Blanquet [34,35]. This involved listing all species usually within an area of 100–400 m^2^ and determining their coverage and abundance, plus noting ecological information (e.g., soils, disturbance, elevation, aspect, and slope). On Robinson Crusoe Island, 106 relevés were made, and 90 were made for Alejandro Selkirk Island. From these data, the computer program TWINSPAN provided an ordination for the assessment of vegetation associations, or zones. For more details of methods, see [32].

The modern vegetation maps reveal dramatic geomorphological (landscape) differences between the two major islands. These differences reflect their age, with the younger island, Alejandro Selkirk, still retaining an oval shape reflecting its volcanic ancestry. At 1 myr of age, erosion has produced deep ravines (quebradas) that slice across the eastern side of the island, some plunging to 500 m. The older island, Robinson Crusoe, at 4 myr of age, has subsided on the eastern-moving Nazca Plate [36,37] and has also been severely eroded, yielding a resistant central basaltic ridge surrounded by broad valleys.

**Figure 4:**
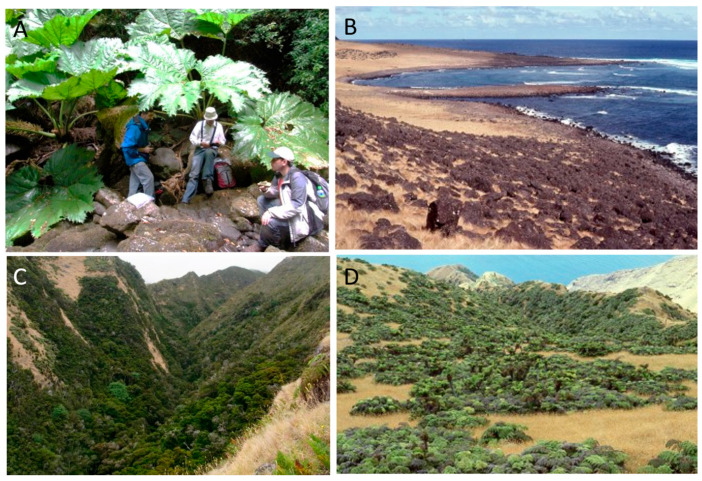
Representative vegetation on Alejandro Selkirk Island. (**A**) Quebrada Casas with *Gunnera masafuerae*; (**B**) coastal grassland; (**C**) middle-elevation *Myrceugenia schulzei* forest; (**D**) *Lophosoria quadripinnata* fern community and grassland at higher elevations.

**Figure 5:**
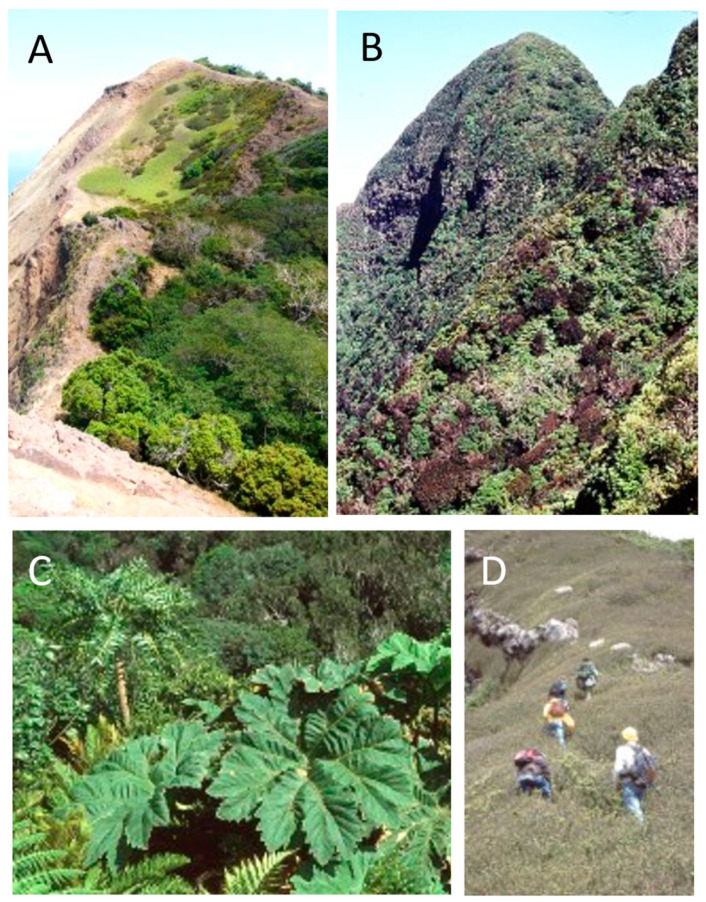
Representative vegetation on Robinson Crusoe Island. (**A**) Edge of upper montane forest (La Pascua) with *Nothomyrcia fernandeziana*, *Drimys confertifolia*, and patches of *Histiopteris incisa*; (**B**) typical upper montane forest (Villagra); (**C**) *Dendroseris pinnata* and *Gunnera peltata* in upper montane forest (Villagra); (**D**) ridge (Salsipuedes) completely covered with the invasive *Ugni molinae*.

The pattern of vegetation zones is different between the two islands (Figure 2, Figure 3, Figure 4 and Figure 5). On Alejandro Selkirk Island, a clear zonation of vegetation can be seen (Figure 2), with grasslands containing many introduced plants near the coast and a fern–grassland mixture or fern communities at higher elevations. In between is the *Myrceugenia* forest, occurring mainly in patches, especially along the steep sides of the quebradas (Figure 4C). Robinson Crusoe Island, in contrast, contains many more vegetation types (Figure 3), largely due to mixtures of native and introduced species (Figure 5). Especially the lower montane forest with *Nothomyrcia fernandeziana* is often invaded by introduced shrubs and herbs. The upper montane forest on this island is dominated by *Drimys confertifolia* with many tall ferns and tree ferns. The western side of the island, including Santa Clara Island, is dry, only becoming green with the spring rains, but this results from persistent introduced grasses and herbs largely of European origin. No clear vegetation bands exist on Robinson Crusoe Island due to its physical alteration from subsidence and erosion over 4 myr and upon which human activities have also played a significant role in the past four centuries.

## 3. History of Human Contacts

Robinson Crusoe Island and Santa Clara Island were first sighted by the Spanish sea captain Juan Fernández on 22 November 1574 [38]. No evidence exists that he went ashore, nor are there published observations of what he may have seen. Word of this discovery was not shared with other European countries, perhaps a deliberate effort by the Spanish authorities to control access. The reason that Juan Fernández located the archipelago was because he tried a new route in sailing from Callao (port of Lima) south to Chile. The traditional route was to sail close to shore, but this meant going against the north-flowing Humboldt Current, which made for a very slow passage. By first sailing westward into the Pacific and then turning south, he made excellent time, so much so that the authorities were suspicious of his claim. Only after other ships had duplicated this same route was Juan Fernández removed from suspicion. It is unclear exactly when and by whom Alejandro Selkirk Island was first discovered [17] (p. 68), but its existence was commonly recognized by late in the 17th century (e.g., Antonio de Vea in 1675) [39].

The discovery of Robinson Crusoe Island quickly led to efforts to establish a colony there, one successfully accomplished by the Spaniard Capt. Sebastián García Carreto in 1591, but which lasted only until 1596 [40]. Another effort took place in 1599 by Martín de Zamora, Diego de Ulloa, and Fernando Álvarez de Bahamonde, also from Spain, and this was somewhat more successful, lasting until the first decade of the 17th century. These colonists brought goats, cultivated plants, and built homes from wood of the native forest [40]; this was the beginning of the human impacts on the ecosystem.

By the turn of the 17th century, other European powers became interested in Robinson Crusoe Island because of its abundant fresh water, numerous fish and lobster, and goat meat from animals that had been left by previous visitors and early colonists. The Dutch were actively exploring the world, and two visitors deserve mention. Willem C. Schouten and Jacob Le Maire, in their ships *Eendracht* and *Hoorn*, made the dangerous passage around South America (resulting in the naming of Cape Hoorn [=Horn]). The *Hoorn* was lost due to fire, but the *Eendracht* finally arrived at Robinson Crusoe Island on 1 March 1616. Schouten [41] published observations from this voyage and mentioned that on the island were fruit trees as well as wild goats and hogs. These reports greatly stimulated other vessels to sail to Robinson Crusoe Island, largely because it offered a convenient place to rest crews and repair ships before continuing further westward, as well as to serve as a lair from which to attack Spanish ships and coastal colonies.

**Figure 6:**
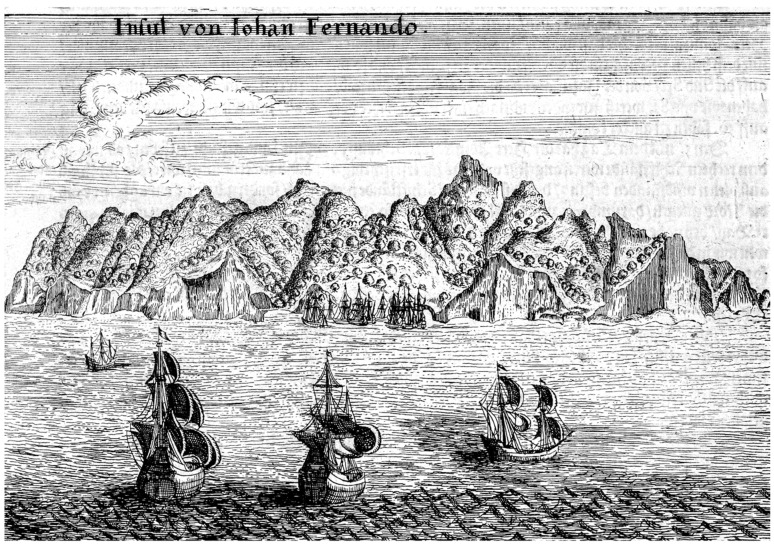
Sketch of the northeastern side of Robinson Crusoe Island. From L’Hermite in Gottfried and Merian in 1631 [43].

Another important early Dutch visitor sailing under the Dutch flag was Jacques L’Hermite from 5 to 13 April 1624. He arrived with a large fleet of 11 ships under support of the East India Company. Adolph Decker, captain of the Marines on this voyage, wrote impressions of their visit to Robinson Crusoe Island [42]. He commented on the many goats, palm trees (the endemic *Juania australis*), a few quince trees, and many sandalwoods. Most impressive was the sketch of Robinson Crusoe Island they made from a ship anchored on the northeastern side of the island (Figure 6); this is a remarkably accurate drawing. A cluster of ships is anchored in Bahía Cumberland (Cumberland Bay), which is the only well-protected bay on the island. The tallest peak is El Yunque and the ridge above the bay is Cordón Central. To its left is Damajuana, with the small cove, El Pangal, at its base. Significant is that trees are shown all along the island, in the central valley, and in the valley to the right, now called Puerto Inglés, which at the present time consists of only introduced herbs and shrubs below c. 350 m. At this period, trees, probably the dominant species *Nothomyrcia fernandeziana*, covered the landscape, being interrupted only by sharp coastal precipices.

**Figure 7:**
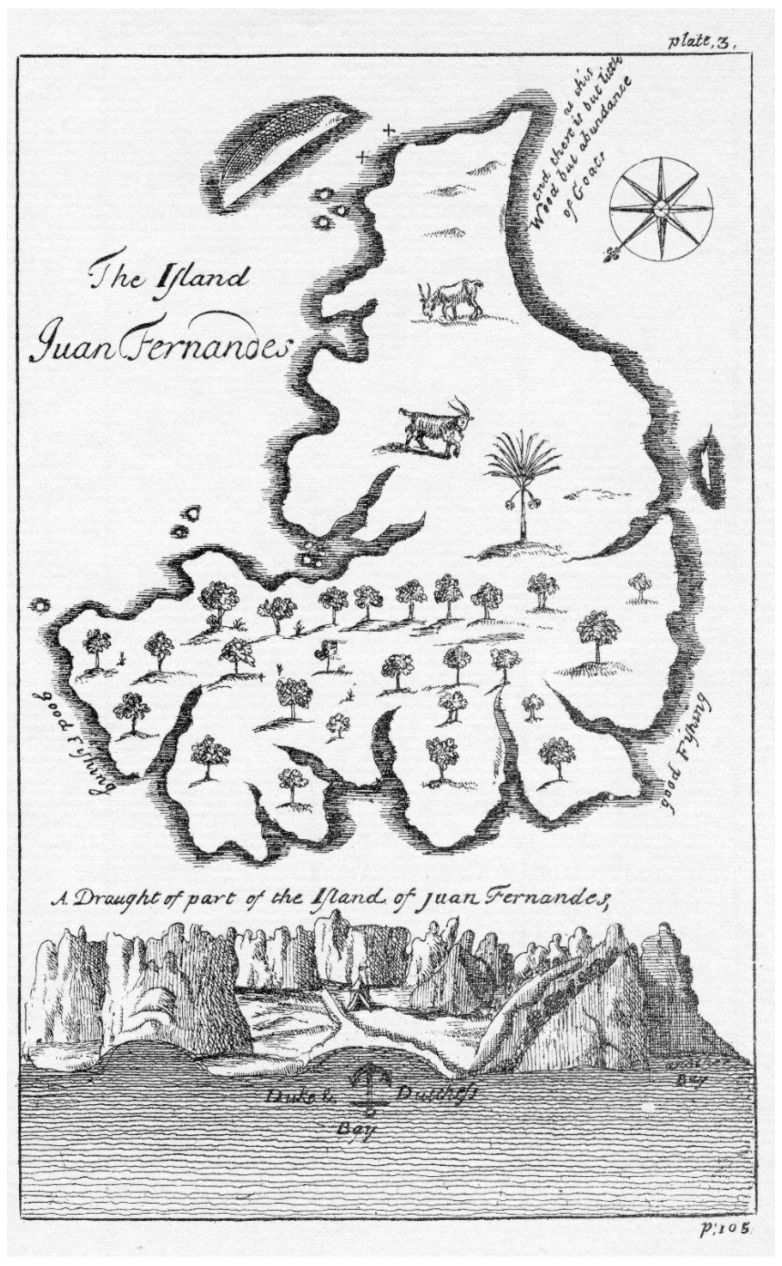
Map of Robinson Crusoe and Santa Clara Islands (**above**) and rough sketch of the northeastern side of the former island. Tree symbols indicate forested areas of the island. From Cooke in 1712 [44].

Toward the end of the 17th and into the middle of the 18th centuries, other visitors came to the Juan Fernández Archipelago, including English navigators, many of whom were privateers. The objective continued to be seeking fresh water, obtaining fresh meat and vegetables, and using it as a place from which to attack Spanish possessions. An example of these adventurers is Edward Cooke, who arrived at Robinson Crusoe Island on 31 January and stayed until 13 February 1709. He stressed the abundance of marine life around the islands, fish, lobster, elephant seals, and numerous fur seals [44]. His sketch of the island (Figure 7), strongly influenced by Ringrose [45] and Funnell [46], shows a much less accurate sketch of Robinson Crusoe Island, but notable is the cover of trees on the map along the northeastern part of the island, in a manner similar to those drawn by L’Hermite.

Another British navigator who visited Robinson Crusoe Island was George Anson. His arrival was filled with tragedy. He sailed from Portsmouth, England, on 18 September 1740 with a squadron of six ships, and after bad weather around Cape Horn, plus illness of the crew, he limped into Robinson Crusoe Island on 9 June 1741 with only three vessels and 50 men (from an original 139). As a result of these misfortunes, Anson stayed on the island until 19 September 1741, more than 3 months. During this time of repair, rest, and recovery, Anson made many observations [47] not only on Robinson Crusoe Island but also on Alejandro Selkirk Island, the first detailed observations of the far island.

**Figure 8:**
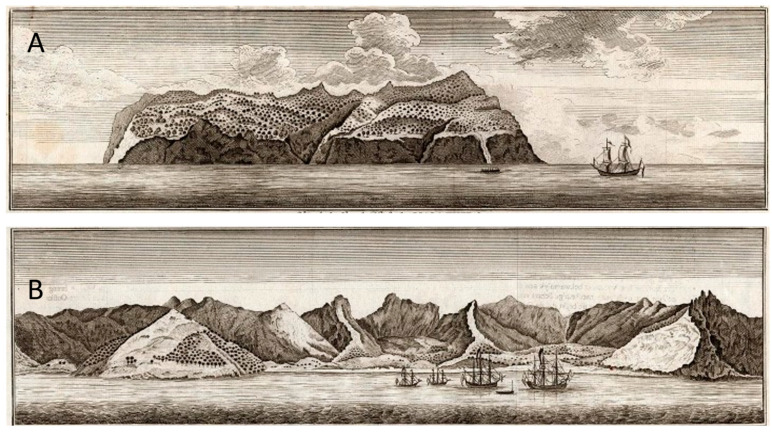
Drawings of islands of the Juan Fernández Archipelago. (**A**) Eastern side of Alejandro Selkirk Island; (**B**) landscape surrounding Cumberland Bay on Robinson Crusoe Island. From Anson et al. in 1748 [47].

Few ships had bothered to stop at Alejandro Selkirk Island simply because it is much further west into the Pacific Ocean (181 km) and because it lacked a suitable protected harbor. Due to its youthful geology (1 myr), the island still retains a cone shape, which means erosion and subsidence have not yet produced broad valleys that would be converted into a safe bay for anchorage. The sea floor on Alejandro Selkirk Island drops quickly downward, posing difficulties for secure anchorage and not offering protection against the wind and waves. Anson’s drawing (Figure 8A) shows the eastern side of the island with a cover of trees all along, interrupted only by seaside cliffs.

**Figure 9:**
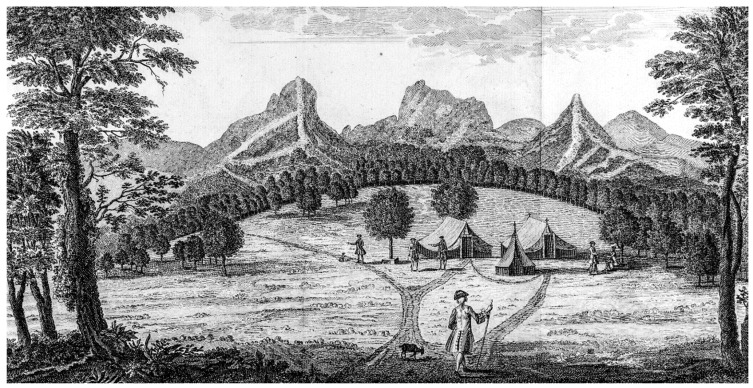
Drawing of George Anson’s campsite in the central valley adjacent to Cumberland Bay, Robinson Crusoe Island. From Anson et al. in 1748 [47].

Also informative are the drawings made by Anson et al. [47] of Robinson Crusoe Island. Figure 8B shows a broad view of the northeastern side of the island in the region adjacent to Cumberland Bay, now revealing some loss of tree cover within the central valley, likely due to the felling of trees in the region by visitors over the past century. Another view of the landscape in the central valley (around Anson’s camp; Figure 9) shows some areas having been cleared of “laurel” trees (probably *Nothomyrcia fernandeziana*) [47] but still with a conspicuous cover of tree vegetation near the shore.

By 1750, Spain had tired of dealing with privateers of various European nations using Robinson Crusoe Island as a lair for harassing her shipping and colonial assets. The decision was made to construct a fort, Santa Bárbara, to ward off unwanted visitors of other nations from arriving in Bahía Cumberland [40]. This objective was achieved along with the establishment of a permanent colony of settlers, called San Juan Bautista, which from that moment onward has been maintained, although the size of the population has ebbed and flowed. With more colonists came the need for wood for houses, the raising of domesticated animals for food, and the cultivation of plants for nutrition and ornament. In addition to the settlers was a military garrison to keep peace, plus convicts from the continent, these ranging from petty thieves to hardened criminals. This mixture of people on an isolated island was not a good recipe for tranquility. Women were scarce, which heightened tension and conflict among the men.

The abundant population of fur seals on Alejandro Selkirk Island, and its lack of protection by Spain, resulted in the development of a massive trade in pelts of these animals for the market in Canton. Whaling and sealing ships, mainly from England and New England (U.S.A.), converged upon Alejandro Selkirk Island from 1792 to 1807 and harvested more than 3,000,000 skins [48]. The animals, having no fear of humans, could be easily killed with a club. This slaughter only stopped when the rapidly declining population no longer generated sufficient profits. During this time, dozens of ships visited the island, sometimes staying for months, which necessitated the building of houses for workers. These activities resulted in the cutting of forests along the eastern side of Alejandro Selkirk Island, which was accelerated when the island was made into a penal colony containing 180–190 convicts in the early 20th century (1909–1930) [17].

After Chilean independence in 1818, more European visitors arrived at Robinson Crusoe Island, and some of these were botanists. These men (and women) were especially interested in the flora and vegetation, which makes their observations even more valuable. The most significant was Claudio Gay. He arrived in Chile from Paris on 8 December 1828 as a professor in the new Colegio de Santiago. Among many excursions to parts of Chile, Gay visited Robinson Crusoe Island on 2 February 1832, apparently staying one month. He quickly prepared a summary of his trip, published in the *El Araucano* [23], which provided many details of the flora and vegetation, including documentation of the numerous introduced plants, especially in the area around the village of San Juan Bautista. He also commented upon the many rats, dogs, and goats.

**Figure 10:**
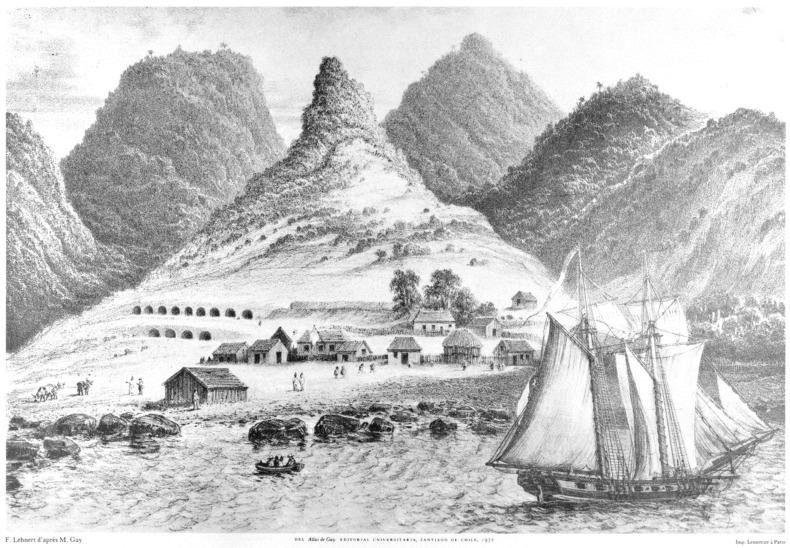
Central valley and the village of San Juan Bautista on Robinson Crusoe Island. Sketch by Claudio Gay in 1832 and published in 1854 [49].

Useful for our discussion is the examination of a drawing by Gay published in 1854 [49], but it was based on sketches made during his trip to Robinson Crusoe Island in 1832 (Figure 10). This shows clearly the strong negative impacts deriving from the colonists and other visitors to the central valley surrounding the village of San Juan Bautista. One can see houses, some unfenced cattle, a few planted large trees, and two rows of caves. These housed political prisoners during the war of independence with Spain, when royalists retook Chile and sent rebels to live in isolation on the island, forced to live in caves they were obliged to carve into the hillside. Most notable is the absence of trees in this part of the island. By the mid-19th century, the native forest had been cut on Robinson Crusoe Island not only in the central valley but now also in the valleys of other places of anchorage, i.e., Puerto Francés, Puerto Inglés, and La Vaquería [17].

**Figure 11:**
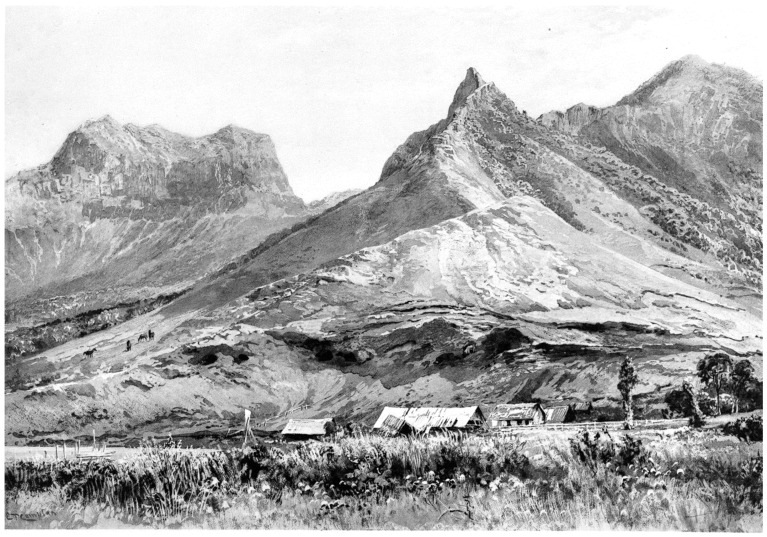
View of the central valley on Robinson Crusoe Island, with the peak El Yunque in the left background and Cordón Central in the center. Note the partially visible caves of the patriots. From Johow (1896: pl. IV) [25].

Federico Johow was another European naturalist, born in Prussia, who received his doctorate from the University of Bonn, Germany, and then departed to Chile as a professor in the new Instituto Pedagógico in Santiago. He was invited by the Chilean government to visit the Juan Fernández Archipelago from 25 December 1891 to 3 February 1892 to prepare a report on its natural and human resources. Johow concentrated on the flora, which resulted in a book in 1896, *Estudios sobre la flora de las Islas de Juan Fernández* [25]. Johow contributed much to understanding the flora and vegetation of all the islands of the archipelago. Of particular interest is his documentation of the high degree of forest clearing in the central valley of Robinson Crusoe Island (Figure 11), which was the situation all along the northeastern side of the island by the end of the 19th century.

One of the strongest contributors to understanding the flora and vegetation of the Juan Fernández Archipelago was the Swedish botanist, Carl Skottsberg. His major expedition to the islands took place from 1 December 1916 to 30 April 1917, the longest stay of any naturalist at that time. He continued to document the endemic and native plant species, with long biogeographical discussions on the origin of the flora [50,51]. He also pointed out the high levels of introduced species (as had Johow), which had taken over many disturbed regions of the island, especially around the village of San Juan Bautista. At this time, the islanders had developed a cattle industry to produce meat for sale to passing ships as well as for the continent. The animals were mostly allowed free range near the village.

**Figure 12:**
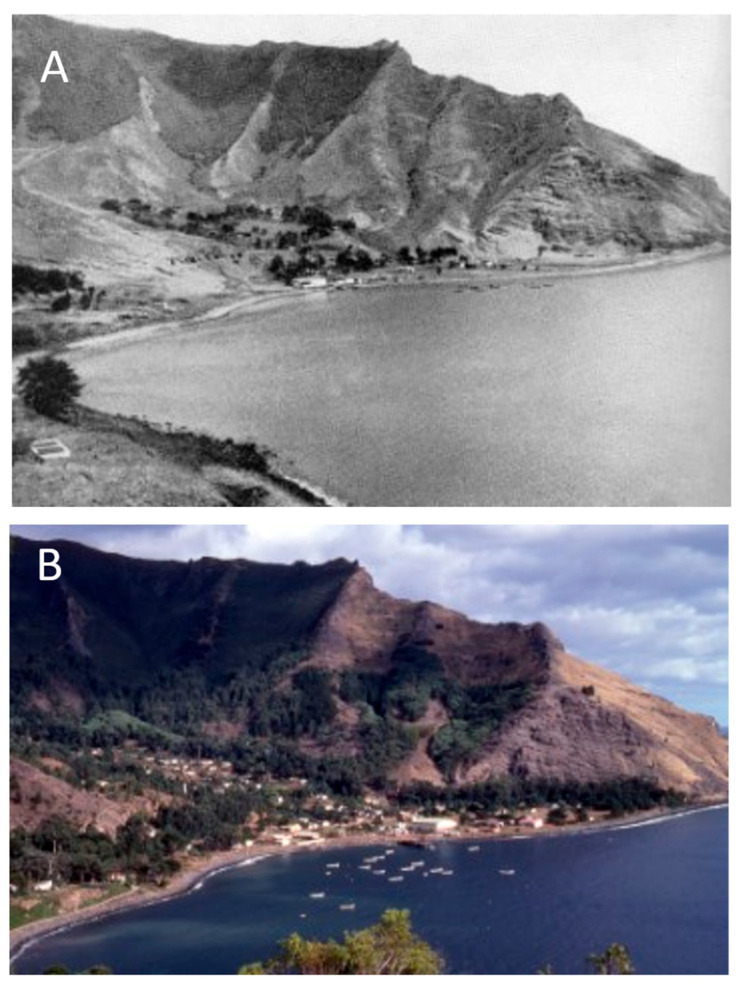
Extent of cover of introduced trees around San Juan Bautista in the central valley on Robinson Crusoe Island. (**A**) From Weber (1940: 33) [52]; (**B**) original photo (1980).

**Figure 13:**
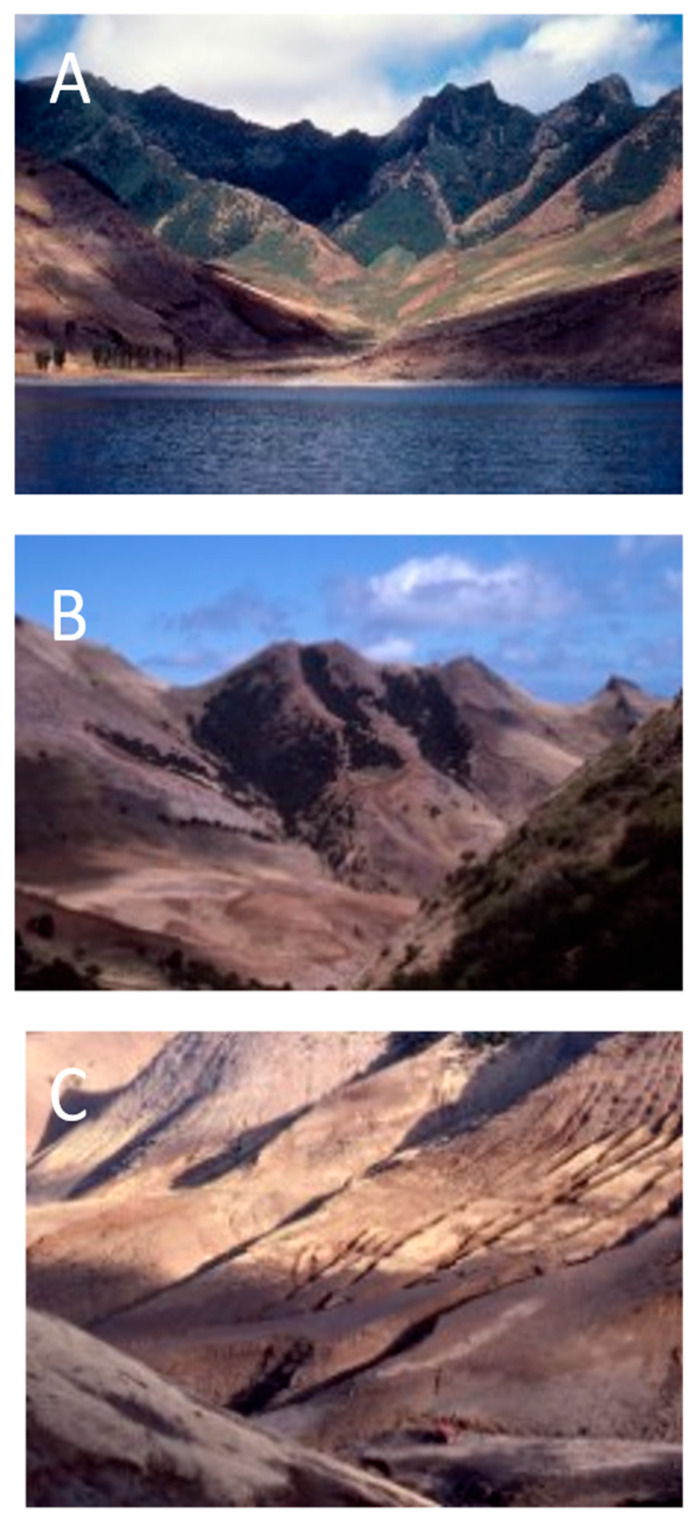
Loss of tree cover in valleys on the northeastern side of Robinson Crusoe Island. (**A**) Puerto Inglés (1980); (**B**) La Vaquería (1990); (**C**) Puerto Francés (1984).

Since the beginning of the 20th century, efforts were made to plant exotic trees in the central valley, especially species of *Cupressus*, *Pinus*, and *Eucalyptus*, in an attempt to hold the soil, provide shade, and also for building materials and firewood. This achieved some success by 1940 (Figure 12A) [52]. These efforts have continued to the present day, now yielding a suitable vegetational cover in the area (Figure 12B). The other valleys along the northeastern side of the island, however, remain largely devoid of trees, especially at the lower elevations (Figure 13).

## 4. Principal Human-Induced Impacts

The most serious human impact on the vegetation of the Juan Fernández Archipelago has been the direct cutting of native forests. On Robinson Crusoe Island, the visits of ships from 1574 to 1750 resulted in the anchorage of vessels not only in Cumberland Bay but also off the shore of Puerto Inglés, La Vaquería, and Puerto Francés. Although these other locations do not provide as much protection from wind and waves as in Cumberland Bay, captains were able to send smaller boats to shore with crews to cut trees for lumber and firewood. Although direct historical evidence is lacking for the building of houses in these other valleys, it is likely that temporary housing would have been constructed for the sailors during their harvesting of trees. The modern vegetation map of Robinson Crusoe Island (Figure 3) clearly shows the eroded areas in these valleys (see also Figure 13) as well as the abundance of assemblages of introduced species.

Impact on the native forests of Alejandro Selkirk Island can also be seen in the modern vegetation map (Figure 2) where the dominant tree, *Myrceugenia schulzei*, is nearly absent from the coastal region, now being confined to slopes at middle elevations adjacent the quebradas. The lower slopes are now covered primarily by introduced grasses such as *Anthoxanthum odoratum*, plus invasive herbs such as *Rumex acetosella* and *Hypochaeris radicata* [53]. Trees were felled on Alejandro Selkirk Island especially during the time of the penal colony (1909–1930), when the convicts were forced to cut trees and prepare lumber for sale by the government and also for use on Robinson Crusoe Island.

Another serious disturbance to the vegetation of the islands of the Juan Fernández Archipelago has come from introduced animals, especially goats, dogs, cats, rats, and pigs. The goats were left on Robinson Crusoe Island at the end of the 16th century and have remained ever since. It is estimated that about 100 exist on Robinson Crusoe Island, but there are perhaps 4000 on Alejandro Selkirk Island [54]. These animals are very sure-footed and can easily find refuge on steep cliffs where people and dogs cannot go. It is possible to shoot goats, but their high rate of reproduction coupled with the rugged terrain on Alejandro Selkirk Island make the challenge of eradication formidable. That only about 100 still survive on Robinson Crusoe Island reflects that over the centuries the villagers have hunted the goats successfully on that island, which has a much simpler landscape. Cattle and sheep have been periodically ranched on both major islands, for meat and wool for islanders and as a source of income from sales to passing ships. Coati mundi, likely introduced as a pet, occurs in the native forest on Robinson Crusoe Island and preys on the eggs of native birds. Rats are also a problem for birds’ eggs, and they also eat tender shoots of species of the native flora. Rabbits were introduced to Robinson Crusoe Island in the 1930s [55], and they have become a serious pest, eating native plants, loosening the soil with their burrowing, and distributing seeds of invasives such as *Acaena argentea*.

**Figure 14:**
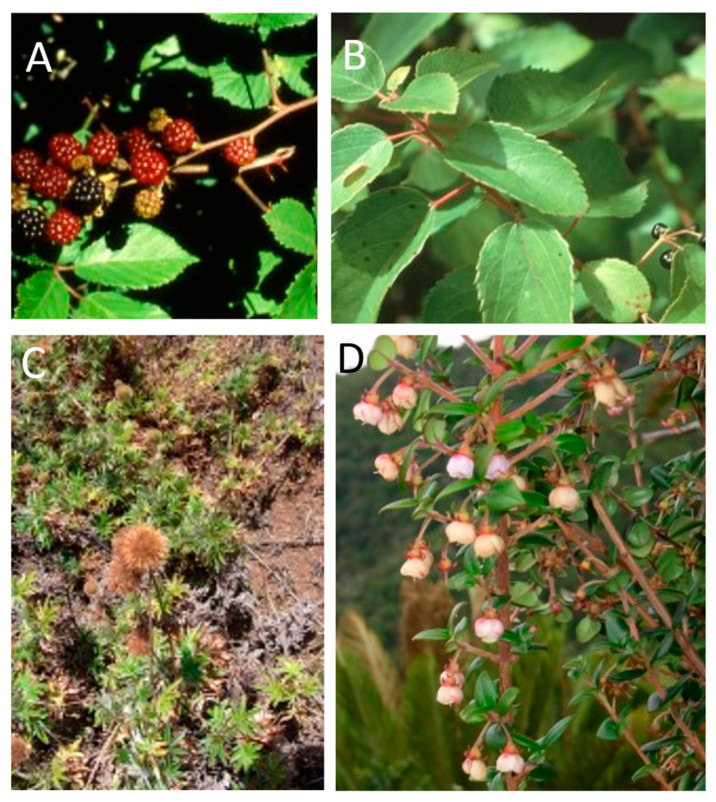
Four major invasive plants in the Juan Fernández Archipelago, especially on Robinson Crusoe Island. (**A**) *Rubus ulmifolius* (zarzamora); (**B**) *Aristotelia chilensis* (maqui); (**C**) *Acaena argentea* (trun); (**D**) *Ugni molinae* (murtillo).

Along with pressure on the vegetation from introduced animals have come pressures from invasive plants. So many species of plants have been introduced since the middle of the 19th century that about one-half of the species of the flora are now of exotic origin [22]. Some invasive species come and go, but four of them have become established across many hectares, especially on Robinson Crusoe Island because of the heavier human presence: (1) *Rubus ulmifolius* (zarzamora; Figure 14A); (2) *Aristotelia chilensis* (maqui; Figure 14B); (3) *Acaena argentea* (trun; Figure 14C); and (4) *Ugni molinae* (murtillo; Figure 14D). The three shrubs, *Rubus*, *Aristotelia*, and *Ugni*, are the most problematical in terms of coverage of Robinson Crusoe Island, combining to occupy 15% of the total land area of the island [32]. *Rubus ulmifolius* has been particularly aggressive, arriving in 1927 and now covering 7% of the land surface [56]. Noteworthy is that most of the serious invasive species have been introduced during the past 150 years [56]. Based on the examination of the environmental parameters on Robinson Crusoe Island, it has been estimated that *Aristotelia chilensis* and *Ugni molinae*, without effective conservation measures, will invade many more areas in the future [56].

Fire has also been a factor that has damaged the native forest on both islands of the Juan Fernández Archipelago. Historical records reveal a series of documented events, some small, but others covering entire valleys (Table 1). One of the most bizarre fires occurred on 19 May 1849, when a group of apparently inebriated California 49ers, on route to the California gold fields, set fire to Valle Inglés, burning down the entire valley! Even more recently, a fisherman’s campfire on Alejandro Selkirk Island escaped and burned 72 hectares of forest [57]. The vegetation apparently recovers from at least some of these fires, but the newly opened areas offer opportunities for erosion and invasive species to colonize and out-compete native elements in reforestation.

**Figure 15:**
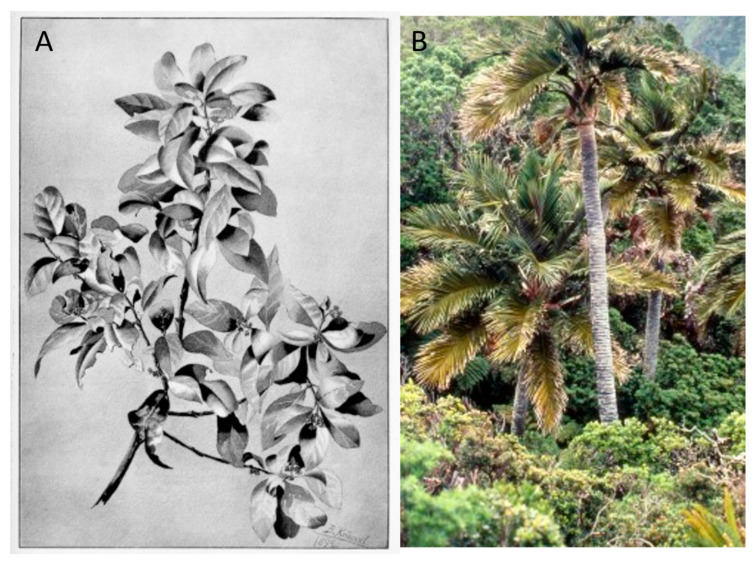
Endemic species on Robinson Crusoe Island that have suffered from extreme harvesting. (**A**) *Santalum fernandezianum*, brought to extinction by 1916 (from Johow, 1896; pl. XIV) [25]; (**B**) *Juania australis*, reduced to c. 1000 individuals.

Several endemic plant species in the Juan Fernández Archipelago have suffered serious population reductions, and most of these have occurred on Robinson Crusoe Island. The most conspicuous example is the extinction of the sandalwood, *Santalum fernandezianum* (Figure 15A). The last tree of this species was observed by Carl Skottsberg in 1908 [60], but when he returned in 1916, it was gone, the wood having been harvested by the villagers of San Juan Bautista [61].

Sandalwood from the Pacific Islands was a thriving commodity in the early 19th century [62]. Hawaii harbored the endemic *Santalum freycinetianum*, but this resource was controlled by King Kamehameha, whereby the species was protected and survived [63]. It is ironic that the species on the far eastern side of the Pacific range of the genus [64], *S. fernandezianum*, far from the main sandalwood harvesting region, was brought to extinction. The strongly scented wood was highly sought in oriental markets and carried a high price, too much so to be resisted by ships passing Robinson Crusoe Island en route to Canton.

Another species that suffered greatly at the hands of sailors of visiting ships to Robinson Crusoe Islands was the endemic palm, *Juania australis* (Figure 15B). What was sought from this species was not the stem or leaves but the edible shoot apex (meristem). When cut off and boiled in water, the tissue tasted like cabbage, hence the popular name “cabbage tree”. One can imagine how good a grilled goat with *Juania* shoot meristem must have tasted to sailors after having been on board ship with a diet of largely dried meat and hard bread. Unfortunately, harvesting the shoot apex requires cutting down the entire tree, which resulted in a serious loss of individuals. It is estimated that now only about 1000 plants survive [65], but luckily these are legally protected and also grow mostly in inaccessible higher ridges or secluded small valleys [66].

A third precarious species, surviving by only a few individuals, is *Robinsonia berteroi*. This is a dioecious species (as are all species of this genus), and it was believed extinct [67], the last plant (male) having died in Villagra valley on Robinson Crusoe Island. A recent ascent and inventory in 2015 of the tallest peak on the island, El Yunque [68], by CONAF (Corporación Nacional Forestal) guides and associates, has revealed another individual. Several specimens collected from 1988 to 1990 and deposited in the National Botanical Garden of Chile have been identified recently as this rare species [69]. More careful searching on the island is needed.

There are several other endemic species of genera (e.g., *Chenopodium, Dendroseris*, and *Nicotiana*) with very low numbers of individuals below any meaningful MVP (Minimum Viable Population) size according to the 50/500 rule of thumb, although there is some discussion about this concept [70,71]. While not directly linked to extinction risk, in conservation, an effective population size (N_e_, roughly the number of reproductive individuals) of 50 is regarded as the lower limit for short-term viability and an N_e_ of 500 is required for long-term viability. In any case, their very small numbers render the island endemics highly threatened and probably in the state of an “extinction debt” [72], pointing to their disappearance in the near future. We have discussed this disappearance of endemics and their replacement by alien plants in the context of an extinction-based saturation currently shaping the island flora of Alejandro Selkirk [73].

The various pressures on the native and endemic plants of the Juan Fernández Archipelago have resulted in a highly endangered vascular flora. Of the 208 species (applying IUCN criteria), 33% are regarded as vulnerable, 40% endangered, 10% critically endangered, and 4% extinct [17] (p. 42). This gives a total of 87% of the species being vulnerable or worse and 50% being endangered or critically endangered. The only conclusion that one can draw from these data is that the native and endemic vascular flora of the Juan Fernández Archipelago are in a fragile condition.

## 5. Assessing and Conserving Genetic Diversity

When human activities reduce population sizes or lead to the loss of some populations, species-wide genetic diversity is lowered, making species more vulnerable to extinction [14,74]. Risk factors include increased inbreeding, vulnerability to stochastic factors, and reduced inter-population gene flow as a result of the extirpation of populations. A loss of genetic diversity reduces the potential for adapting to future environmental changes resulting from climate change, habitat disturbance, etc., associated with human activities.

Various genetic markers (e.g., allozymes, ISSRs, AFLPs, and microsatellites) have been used in population genetic and phylogenetic studies of plants on Juan Fernández, with implications for conservation [21,75,76,77,78]. These studies were carried out over several decades and provided valuable insights into the level and apportionment of genetic variation within and among populations of numerous endemic species [79]. The markers are also useful for assessing genetic divergence among congeneric species and for inferring possible modes of speciation [80]. Our prior genetic molecular studies were valuable for the conservation of genetic diversity in Juan Fernández endemics; however, the prior markers were limited because they resolved a relatively small number of loci, with a percentage of the loci being invariant [81]. These two factors reduce the utility of the markers for studies of island plants where genetic variation is typically lower than in continental taxa [82].

Although previous studies of genetic variation using allozymes and other population-level markers have been useful in the Juan Fernández flora, next-generation sequencing (NGS) that generates thousands of markers (single nucleotide polymorphisms, SNPs) throughout the genome provides a better estimate of diversity within populations and species than was obtained with prior markers [74,81]. There is now increased interest in genomics and its application to conservation in island plants [83,84,85,86], including resolving phylogenies [87,88] and inferring mating systems [89,90]. A variety of methods are increasingly available for generating sequences, and costs continually decrease [85,86,91]. Particularly noteworthy is the assembly of a reference genome for an island endemic species [92,93]. Genome assembly, however, is rather labor-intensive; many steps are required from the extraction of DNA to base calling and the identification of SNPs, let alone genome assembly. For Juan Fernández plants, a feasible approach for producing genomic markers (SNPs) would be reduced representation sequencing, such as RADseq [86]. An important advantage of NGS studies is that smaller (3–5 mg fresh weight) and fewer samples are required, because many more markers are generated per plant.

### 5.1. Genetic Diversity and Divergence in Rare Taxa

**Figure 16:**
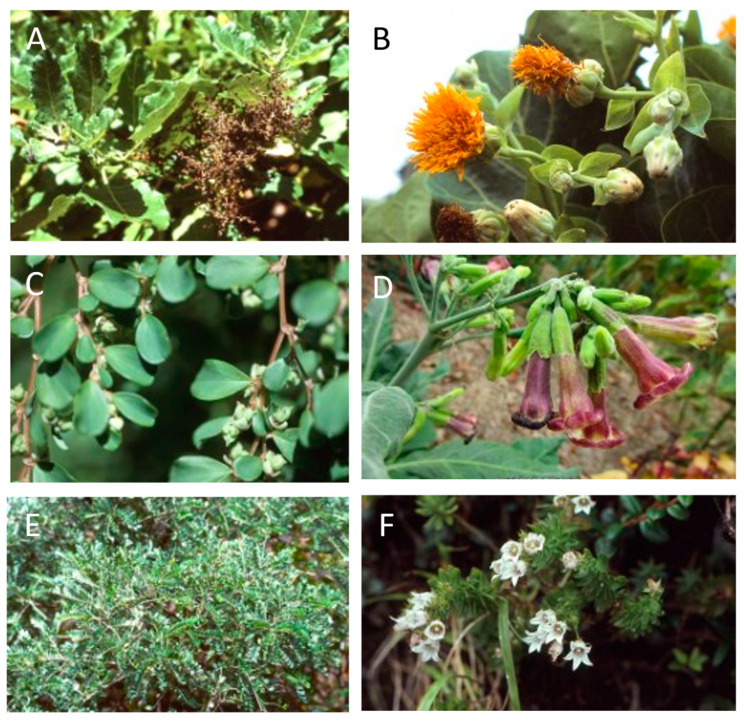
Genera containing endemic species in Robinson Crusoe and Santa Clara Islands, Juan Fernández Archipelago. (**A**) *Chenopodium sanctae-clarae*; (**B**) *Dendroseris litoralis*; (**C**) *Lactoris fernandeziana*; (**D**) *Nicotiana cordifolia*; (**E**) *Sophora fernandeziana*; (**F**) *Wahlenbergia fernandeziana*.

To illustrate the potentials and opportunities with genomic data for informing conservation of Juan Fernández endemic plants, we review several genera (Figure 16) on Robinson Crusoe and Santa Clara Islands: *Chenopodium* (Chenopodiaceae); *Dendroseris* (Asteraceae); *Lactoris* (Lactoridaceae); *Nicotiana* (Solanaceae); *Sophora* (Fabaceae); and *Wahlenbergia* (Campanulaceae). Species in these genera have suffered habitat reduction and loss of populations due to landscape modifications from island subsidence and erosion over four million years, overlain by intensive human impacts including the activities of the introduced animals (browsing, seed dispersal of alien plants) over the past three centuries. It is not possible to infer exactly which human activity contributed to a reduction in population size or number within a particular endemic species, but the overall impact has been negative.

*Chenopodium*: Three endemic species in the Juan Fernández Archipelago are known from a total of four populations and an estimated 60 individuals in nature [65]. Allozyme markers failed to resolve variation in the two species on Robinson Crusoe and Santa Clara Islands (*C. crusoeanum* and *C. sanctae-clarae*, respectively) [21]. More variable markers are needed to assess diversity within and between these two morphologically similar species [61].

*Dendroseris*: With 11 species, this is the largest endemic genus in Juan Fernández [18]. Four species have fewer than 5 plants from 1 or 2 populations, two species consist of ca. 50 individuals from 3 and 6 locations, three species have ca. 100 plants (2 from 8 populations and 1 from 12), and two species have fewer than 500 plants (12 and 18 locations) [65]. It is remarkable that this largest radiation in Juan Fernández is now represented by only ca. 1200 plants in nature [65]. Genomic markers would be invaluable for assessing genetic diversity within this remarkable genus, and these data would be critical for formulating conservation strategies.

*Lactoris*: The endemic family, Lactoridaceae, represented by *Lactoris fernandeziana*, has been the subject of a variety of studies on reproductive biology, conservation [94,95,96], and genetic variation [97,98]. However, several issues of important conservation concerns remain. Once thought to be extinct or nearly so [99], Ricci [96] estimated ca. 1000 plants (over 30 cm in height) in 14 populations at an elevation of 450 m or higher on Robinson Crusoe Island. Determining how genetic variation is structured geographically in *Lactoris* across the island would be informative for conservation purposes. Important issues for local populations include the levels of diversity in populations of different sizes, which vary from fewer than 10 to ca. 250 individuals [96], and whether the mating system in this self-compatible species [95] varies with population size.

*Nicotiana cordifolia* subsp. *cordifolia* and subsp. *sanctaclarae*: The typical subspecies is known from several populations consisting of possibly fewer than 50 plants on Alexander Selkirk Island. Based primarily on flower color, subspecies *sanctaclarae* was described by Danton [100] from Santa Clara Island. Estimates of genetic diversity within populations and divergence between the two subspecies would be of value for the conservation of genetic diversity in the species.

*Sophora fernandeziana* var. *fernandeziana* and var. *reedeana*: This species (both varieties) is represented by possibly fewer than 10 populations [65]. Bernardello et al. [101] discussed the vulnerability of these taxa. Allozyme diversity within five populations was very low [21]; more variable markers are needed for assessing genetic variation in this rare species.

*Wahlenbergia*: One question of conservation interest is whether the two species on Alexander Selkirk Island, *W. masafuerae* and *W. tuberosa*, are distinct genetic entities as suggested by Lammers [102] or a single variable species. Areas of spatial overlap should be examined because Lammers [102] indicated several mixed collections in herbarium material. It is important to note that some populations occur at sea level and are potentially vulnerable to future sea level rise. A second important conservation issue in *Wahlenbergia* involves assessing the genetic diversity within and the structure among the few small populations of *W. berteroi* at different elevations on Robinson Crusoe Island and on Morro Spartan, a rock off the coast of Santa Clara Island. Like *Wahlenbergia* on Alexander Selkirk Island, several populations of *W. berteroi* occur near sea level.

### 5.2. Role of Mating Systems

Extensive surveys of the reproductive biology of Juan Fernández plants were carried out by G. J. Anderson, G. Bernardello, and collaborators [103]. However, despite their exemplary studies, little is known about the mating systems of endemic species, that is, who mates with whom and in what frequencies. The mating system can vary from highly selfing (low or no outcrossing) to highly outcrossing. A combination of partial selfing and partial outcrossing results in a mixed mating system [104]. The mating system is important in shaping the level and pattern of genetic diversity in species, and this is especially critical in small island populations. High selfing will result in low genome-wide genetic diversity of plants within populations [105] and high divergence among populations. Conservation of the maximum genetic diversity in selfing species would entail conserving multiple populations, whereas in outcrossing species, diversity could be captured in one or a few populations. Determining mating systems in the species consisting of small, scattered populations characteristic of many Juan Fernández taxa would be extremely valuable for informing strategies for conserving genetic diversity within species. The small amounts of material required for obtaining a large number of markers and for inferring mating systems with high confidence are major advantages for studying rare insular species [89,106].

Anderson et al. [107] conducted an elegant study of the breeding systems of several species of *Wahlenbergia*, combining floral morphology with field experiments. However, nothing is known about the mating system of the species. Especially intriguing is the Anderson et al. [107] hypothesis of a very unusual mechanism for possible partial autogamous selfing in the rare species, *W. berteroi*, on Robinson Crusoe Island. It is inferred that pollen is first deposited on the inner surface of corollas by the “pollen brush” followed by transfer to the stigmas by wind. Genomic markers could determine how the unusual floral morphology of *W. berteroi* shapes the mating system of the species, which would be useful in formulating strategies for the conservation of genetic diversity. Another endemic species, *W. fernandeziana*, occurs in a range of population sizes and over a range of altitudes on Robinson Crusoe Island. Like *W. berteroi*, it appears to be basically self-compatible, but the seed set varies within the species.

Information on the mating system, indicating whether it varies with population size, altitude, or habitat, would be of value for conserving the maximum genetic diversity in *W. fernandeziana*.

### 5.3. Genomic Markers and the Botanical Garden

**Figure 17:**
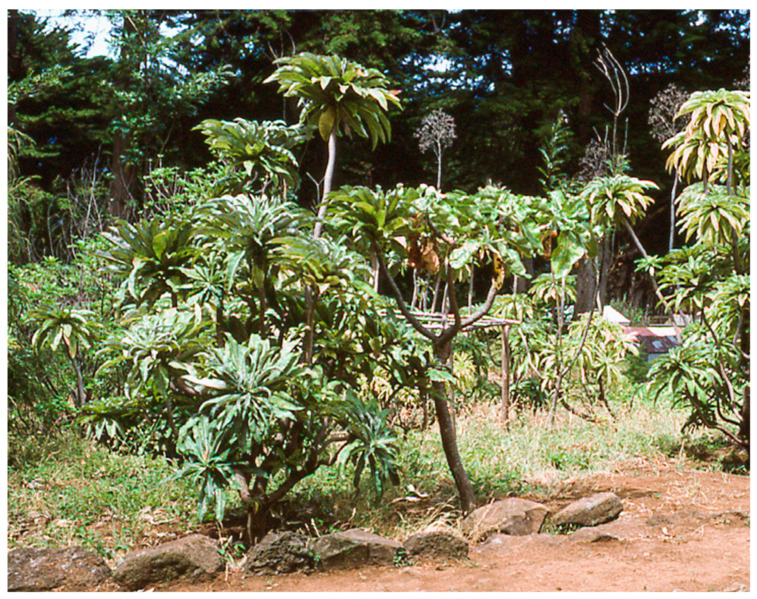
Botanical garden on Robinson Crusoe Island, administered by the Corporación Nacional Forestal (CONAF).

Ricci [65] listed 79 endemic species growing in the CONAF garden (Figure 17). The garden is a valuable resource for the conservation of plants, particularly when species are extremely rare or even extinct in nature. In addition, the reproductive biology of plants may be more conveniently studied in cultivation than in isolated, inaccessible natural populations [103]. An important general caveat with plants in cultivation is that when normally ecologically spatially isolated, interfertile species are brought together, and hybridization may occur and result in the loss of “pure” species [108]. Genomic markers can serve as a sensitive method for detecting hybridization, and several examples of putative hybridization in the CONAF garden and village may be cited.

*Chenopodium*: Two species of *Chenopodium* are in cultivation on Robinson Crusoe Island. One species, *C. sanctae-clarae*, is known only from a single population on Morro Spartan, a rock off the coast of Santa Clara Island. Luckily, this species has also been successfully cultivated in the village as an ornamental, which provides conservation protection. *Chenopodium crusoeanum* is known from two small populations on Robinson Crusoe Island. Neither species exhibited allozyme variation, but the two species were not compared directly to each other [21]. Seeds collected from multiple plants in the natural population of *C. sanctae-clarae* had higher, more rapid germination than seeds from a single plant in cultivation [109]. Genomic markers could provide insights into the possible reason(s) for the difference in seed quality and inform conservation strategies for these two rare species. The lower seed germination from the single plant could be due to inbreeding or possibly hybridization with the other cultivated species, *C. crusoeanum*. Diversity in the natural population of *C. sanctae-clarae* could also be used as a guide for the conservation of plants on Morro Spartan.

*Dendroseris*: Morphological observations suggest extensive interspecific hybridization between species of *Dendroseris* in the CONAF garden and in the village [110,111] (pers. observ.). Markers in cultivated plants could be compared to “pure” species in nature to detect hybrids. This information could inform on whether hybrid plants should be eliminated or otherwise isolated from further gene exchange, and documentation of hybridization should prompt consideration of methods to prevent further hybridization among ostensibly “pure” species in cultivation. Hummingbirds are the mostly likely biotic pollination agents for cultivated plants, with wind being the common abiotic pollen vector [103].

## 6. Conservation Recommendations

CONAF has already undertaken numerous conservation measures to protect the flora of the archipelago, especially on Robinson Crusoe Island. Livestock are now strictly controlled in number as well as restricted to fenced areas at lower elevations where only introduced herbs occur (e.g., Valle Inglés). Periodical eradications of patches of zarzamora (*Rubus ulmifolius*) and maqui (*Aristotelia chilensis*), especially around the village of San Juan Bautista, have helped to limit further spread of these very aggressive invaders. Wooden barriers have been built across small gullies to stem soil erosion that is frequent during spring rains. These initiatives have all been appropriate, but they must be maintained consistently into the future; financial resources are often the limiting factor.

In view of the challenges facing the endemic flora and vegetation of the Juan Fernández Archipelago, we offer some recommendations for consideration. Many suggestions have been published previously [30,51,112,113], but the threatened and endangered status of much of the flora supports once again making pleas for preserving this important natural laboratory of biodiversity and plant evolution.

The first obvious recommendation is for continued vascular plant inventorying of both the Robinson Crusoe and Alejandro Selkirk Islands. Despite the fact that much collecting and publishing on the flora has occurred over the past centuries, and that the rate of discovery of novelties has dropped off substantially, new taxa are still being uncovered (e.g., *Gleichenia lepidota* [114]; *Robinsonia saxatilis* [100]; and *Carex stuessyi* [115]). A very positive development is that with more field observations by the CONAF guides [65], new species (e.g., *Centaurodendron schilleri* [116]) as well as more individuals of rare taxa (e.g., *Robinsonia berteroi* [68,69]) have been found.

A complement to these new observations would be the further development of an herbarium in the CONAF headquarters on Robinson Crusoe Island. A small herbarium now exists, largely due to mounted specimens being donated by previous researchers in the archipelago, but this might be increased. Such an herbarium resource serves as an educational tool for the CONAF guides, but it can also be used for displays and workshops for the inhabitants of San Juan Bautista, as they further develop ecotourism initiatives.

The activities of CONAF in conserving endemic plants of the archipelago in situ, i.e., in the islands, must be supported. The environment of the islands is proper for such cultivation. Already, CONAF has had success with the propagation of seeds of many rare species of the flora, cultivating these in the administrative garden on Robinson Crusoe Island [65], and setting out plants in common areas of the village. Such plantings are attractive for ecotourists and also serve to educate the villagers on the natural heritage of the island.

As mentioned above, a caution with any botanical garden is the propensity of closely related species to hybridize when they are brought into proximity [108]. This is a challenging issue, because it makes sense to preserve rare taxa by cultivating them in a local garden. Because of the costs involved, these gardens are nearly always “common” or open, that is, the species grow close to each other and are open to the environment, including to pollinators. The danger is that hybrids may form, seeds are collected, and then hybrids are inadvertently reintroduced to the wild. Hybrids have been detected, for example, between species of the endemic genus *Dendroseris* [110,111]. Close watch on the morphology is one useful step, but another is to develop means for genetic testing, including genomic markers when necessary to distinguish closely related species and to detect hybrids.

For precision in detecting hybrids and to set conservation priorities for the Juan Fernández flora, a mechanism for evaluation of genetic variation within and among populations needs to be established. This might be developed on Robinson Crusoe Island at the CONAF headquarters, as a separate small laboratory, or it could be conducted in collaboration with a continental lab. Given the isolation of the archipelago and the many challenges of establishing a laboratory in situ, the best approach for genomic conservation studies might be collaboration with research-active genomic laboratories on the continent, such as at the University of Concepción. These collaborations should include the training of graduate students or postdoctorates dedicated to the study of Juan Fernández plants. Such studies would be a natural extension of the more than forty years of collaboration between the faculty of the Department of Botany in Concepción and CONAF in the archipelago [117]. A major challenge of studies on Juan Fernández is accessing natural populations, but this problem is ameliorated by the experienced CONAF guides who know the flora and are capable of getting into highly inaccessible populations. Specimens, either seeds or leaves, could be collected in silica gel and sent to the continent for analysis. Sources of funding, especially for the lab costs, would have to be institutionalized. Once the spectrum of genetic variation is known for a species, it becomes possible to estimate its vulnerability in the face of environmental change. The more genetic variation that a species harbors, the more likely it is that it will be able to survive any environmental perturbation (e.g., temperature shifts, changes in precipitation, flooding, introduced herbivores, or pathogenic microbes).

Collaboration of a genetic laboratory with the park service would not only encourage studies on garden plants but also the study of hybridization in the natural vegetation. It has long been known that hybrids occur between *Gunnera bracteata* and *G. peltata* in Villagra valley on Robinson Crusoe Island [118], but the dynamics of this intermixing are unknown (i.e., are there also advanced generations and/or introgressants?). Another question might be the following: Are the related species *Ugni selkirkii* (endemic) and *U. molinae* (introduced) hybridizing on Robinson Crusoe Island? If so, what are the implications for conservation of the endemic species?

As the economy of Robinson Crusoe Island turns more toward ecotourism, due to the decline in the lobster industry after decades of overfishing [119], it is imperative that the next generation of the villagers in San Juan Bautista learn about the flora and vegetation, respect it, and use this knowledge for teaching visitors to the island. This means teaching children in primary school about the fascinating rare plants. This can be conducted through photographs, some herbarium materials, and field trips. CONAF can help train teachers in developing modules of instruction to increase ecological and conservation awareness. As the CONAF guides are also villagers, they can visit the school and make presentations appropriate for the grade level.

And finally, and very significantly, there must be the development of a phytosanitary inspection system. It does little good to fight hard for the conservation of the endemic species of the archipelago when an influx of new invasive species, especially plant invaders, keeps coming. Traffic to Robinson Crusoe Island, the major point of arrival to the archipelago, comes by boat (usually once or twice per month) or by 5–9-seater planes (often every day, depending upon weather). A comprehensive inspection program could easily be established in the village as people and goods come off the arriving ships. Initially, this will be regarded as an inconvenience and result in some delay for the offloading of cargo, but the villagers must be convinced through discussion and education that it is in their long-term interests to cooperate. Arrivals at the airstrip at the far western side of the island would also have to be inspected. This is completely feasible, but at least one CONAF representative would have to be stationed there every day. It is only by careful monitoring of incoming materials that invasive species can be intercepted, preserving the native vegetation for enjoyment and study by future generations.

## Figures and Tables

**Table 1:** Historical records of fire on Robinson Crusoe Island, based on data in [40] and other historical sources [58] (p. 251).

Date	Circumstance	Location
26 November 1795	Ramón Negrete, Francisco Clavel, Pedro José Gutiérrez, and Marcel Boza set fire after climbing peak	Summit of El Yunque; burned for 8 days
5 January 1816	Apparently an accidental fire set in Chaplain’s huts	Began in Chaplain’s huts and spread over “the entire island”
18 November 1837	Peruvians set fire	Burned down village of San Juan Bautista
19 May 1849	20 Californians set fires	Entire valley behind Puerto Inglés
25 March 1862	Accidental fire set by Rengifo party	Huge forest fire in Puerto Francés
18 May 1869	Lumberjacks of Fernández López	“Frequent” forest fires in unspecified locations
February 1872	Sailor carelessly sets fire	1 mi^2^ (2.6 km^2^) of good timber burned in unspecified location
1905	Circumstance unknown [51] (p. 108)	El Rabanal (between Valle Colonial and Puerto Francés)
1930	Circumstance unknown [59] (p. 38)	Valle Inglés

## Data Availability

Data are contained within the article.
